# Associations between fine particulate matter and in-home blood pressure during the 2022 wildfire season in Western Montana, USA

**DOI:** 10.1088/2752-5309/add616

**Published:** 2025-05-21

**Authors:** Ethan S Walker, Taylor Stewart, Rajesh Vedanthan, Daniel B Spoon

**Affiliations:** 1 School of Public and Community Health Sciences, University of Montana, Missoula, MT, United States of America; 2 Department of Population Health, NYU Grossman School of Medicine, New York, NY, United States of America; 3 Providence Heart Institute, Providence St. Patrick Hospital, Missoula, MT, United States of America

**Keywords:** PM_2.5_, indoor air quality, BP, environmental epidemiology, cardiovascular health

## Abstract

Wildfires continue to increase in size, intensity, and duration. There is growing evidence that wildfire smoke adversely impacts clinical outcomes; however, few studies have assessed the impact of wildfires on household air quality and subclinical cardiovascular health indicators. We measured continuous indoor and outdoor fine particulate matter (PM_2.5_) concentrations from July–October 2022 at 20 residences in the rural, mountainous state of Montana in the United States. We used a combination of satellite-derived smoke plume data from the National Oceanic and Atmospheric Administration’s Hazard Mapping System and household-level daily mean PM_2.5_ concentrations to classify wildfire-impacted days. One participant from each household self-reported in-home blood pressure (BP) on weekly electronic surveys. We used linear mixed-effects regression models to assess associations between air pollution exposures (PM_2.5_ concentrations; number of wildfire-impacted days) and systolic BP (SBP) and diastolic BP (DBP). Models were adjusted for potential time-variant confounders including temperature, humidity, and self-reported exercise. Compared to survey periods with 0 wildfire days, SBP was 3.83 mmHg higher (95% Confidence Interval [95% CI]: 0.22, 7.44) and DBP was 2.36 mmHg higher (95% CI: −0.06, 4.78) during periods with 4+ wildfire days. Across the entire study period, a 10 *µ*g m^−3^ increase in indoor PM_2.5_ was associated with 1.34 mmHg higher SBP (95%CI: 0.39, 2.29) and 0.71 mmHg higher DBP (95% CI: 0.07, 1.35). We observed that wildfire-impacted days and increasing household-level PM_2.5_ concentrations are associated with higher in-home BP. Our results support growing literature which indicates that wildfires adversely impact subclinical cardiovascular health. Clinical and public health messaging should emphasize the cardiovascular health impacts of wildfire smoke and educate on exposure-reduction strategies such as indoor air filtration.

## 1. Introduction

Elevated systolic blood pressure (SBP) is the second leading risk factor of disease burden globally [1], putting individuals at risk for numerous adverse cardiovascular outcomes such as ischemic heart disease, heart failure, and stroke [2–6]. Measured by disability-adjusted life-years (DALYs; the sum of years of life lost to premature mortality and years lived with disability) [1], the global disease burden of elevated blood pressure (BP) has increased as the rate of both elevated SBP and deaths associated with SBP have grown substantially since 1990 [5]. As a result of the increasing global prevalence of elevated BP, the economic burden of elevated BP and hypertension is staggering in terms of both direct health costs and indirect impacts on employment and productivity [7–9].

The leading risk factor of global disease burden is particulate matter air pollution [1]. Exposure to fine particulate matter air pollution (PM_2.5_; airborne particles ⩽2.5 *µ*m in aerodynamic diameter) is associated with elevated BP as well as other cardiovascular disease outcomes [10–13]. On a global scale, air pollution is the largest environmental risk factor for premature death, with over 6 million deaths attributable to air pollution exposures from indoor and outdoor origin each year [14]. Outdoor air pollution, from sources such as traffic, industry, and wildland fires and other biomass burning, is often the focus of scientific research and air quality guidelines. However, measuring and improving indoor air quality is critically important. In the United States (US), adults spend around 90% of their time indoors [15], where air quality is adversely impacted by air pollutants originating from numerous indoor and outdoor sources, including cooking, biomass burning, and ambient air pollutants that infiltrate indoors [16–18]. Globally, household air pollution from biomass burning for cooking and heating purposes impacts approximately 3 billion people and is one of the leading environmental risk factors for premature morbidity and mortality [19]. In short, air pollution is ubiquitous, as nearly every individual on earth is exposed to some form of air pollution, with lower-income countries and communities bearing the brunt of this burden [14].

While some sources of outdoor air pollution have decreased in recent decades due to improvements in policy and technology, air pollution from wildfires is increasing and will continue to do so due to climate change [20–23]. The burned area from wildfires in the US has nearly quadrupled over the past 40 years, and the contribution of wildfires to overall PM_2.5_ in the US has increased, with up to 50% of ambient PM_2.5_ in some Western regions now attributed to wildfires [22]. With increasing extreme heat and drought events under climate change, these trends are expected to continue in the coming decades [20, 22]. In Montana, a Western US state where this research took place, wildfire season is now 40 d longer than it was just 30 years ago [24, 25]. Even in the absence of local wildfires, smoke from regional or distant fires often travels hundreds of miles and impacts health after long-range transport [26].

There is increasing evidence that wildfire-related air pollution adversely impacts all-cause mortality and cardiovascular and respiratory disease and mortality [27–33]. However, many of the health outcomes studied previously have been clinical in nature. Due to the difficulty of studying wildfires in field settings, few studies have assessed how wildfires impact subclinical health and important risk factors for morbidity and mortality such as BP. Previous research has established likely mechanisms through which particulate matter air pollution impacts health, including pathways of inflammation and oxidative stress, autonomic nervous system imbalance, and direct transmission of air pollution constituents into systemic circulation [11]. These pathways can lead to acute and chronic increases in BP through repeated exposures that increase vascular dysfunction as well as the progression of chronic disease such as atherosclerosis [11]. Wildfires and other biomass air pollution sources have a different chemical composition compared to air pollution from urban areas and may have differential impacts on health [34]. In order to understand the burden of increasing wildfires and to better frame public health and clinical messaging on risk-reduction strategies towards at-risk populations, further research is needed to understand how increasing wildfire air pollution impacts BP.

To help inform these gaps in the literature, our aim was to assess indoor and outdoor household-level PM_2.5_ and in-home BP over the course of the 2022 wildfire season in the wildfire-impacted state of Montana in the US.

## 2. Methods

### 2.1. Study setting and participants

This study was undertaken in the communities in and around the US city of Missoula in the state of Montana. With an estimated population of 78 000 in 2023, Missoula is one of the largest cities in the largely rural state of Montana, which is ranked 4th in land area and 43rd in population (1.1 million residents) out of the 50 US states as of 2023 [35]. Western Montana commonly experiences waves of wildfire smoke each year from both local and regional wildfires. The mountainous terrain in the region also amplifies the impact of wildfire smoke in valley communities such as Missoula, where atmospheric pressure inversions often trap air pollution in low-lying areas for extended periods, leading to poor air quality throughout the year. According to the 2024 State of the Air report by the American Lung Association, Missoula is ranked 14th worst for 24 h particle pollution out of 223 US metropolitan areas [36]. Montana has the highest fraction of mortality attributable to wildfire PM_2.5_ of any US state [37].

We recruited a single adult participant from each of 20 households. Participants were recruited on a rolling basis during June and July of 2022 with the help of a local climate advocacy organization called Climate Smart Missoula. Eligibility criteria included adults (⩾18 years of age), non-smoking and living in a non-smoking household, access to an electronic device to submit online surveys, and living within range of a cellular data tower so a Wi-Fi hotspot could be used in their home. The study was approved by the University of Montana Institutional Review Board, and participants signed a written informed consent form prior to engaging in any study activities.

All study activities were conducted remotely and were coordinated by University of Montana personnel. Equipment was mailed to participant households and included a portable upper-arm BP monitor (BP5350, Omron Healthcare, Inc.) and 2 optical air pollution sensors (PAII-SD, PurpleAir, Inc, USA) that were paired with a Wi-Fi hotspot (Solis Lite, Skyroam, Inc, USA). After 4 months of participation, participants mailed equipment back to the University of Montana using pre-paid postage and were compensated for time spent completing study procedures.

In a prior publication from this study, we further describe study PM_2.5_ concentrations and associations with household and demographic characteristics [16]. We found that median daily indoor PM_2.5_ in study households was over 4 times higher during wildfire-impacted periods compared to non-wildfire periods (2.1 *µ*g m^−3^ vs 10.4 *µ*g m^−3^) [16]. Indoor PM_2.5_ concentrations and infiltration efficiency (the proportion of outdoor PM_2.5_ that infiltrates indoors and remains suspended) varied by household subgroups including household income, age of the home, and presence of air conditioning units and portable air cleaners [16]. We have also published results on wildfire exposures and participant-reported activities [38]. We found that as outdoor PM_2.5_ increased, participants were less likely to report outdoor exercise or that they opened windows in their home for ventilation [38]. The analysis and results described below build off this previous work and describe associations between PM_2.5_ and in-home BP.

### 2.2. Exposure assessment

In-depth methods and results on PM_2.5_ exposure assessment have been published previously [16]. We used PurpleAir sensors to measure indoor and outdoor PM_2.5_, relative humidity, and temperature at 2 min intervals at each household over the duration of the study. Prior to deploying the PurpleAir sensors to study households, we collocated each sensor with a reference monitor (BAM 1020, Met One Instruments, Inc., USA) to ensure all sensors were functioning properly [16].

PurpleAir sensors were mailed and set up at study households on a rolling basis after each participant was enrolled in the study. After participants received their study equipment in the mail, placement and setup of PurpleAir sensors was guided by written instructions and through a setup call with study personnel. Indoor sensors were placed in a common room where participants spent a majority of their time while at home. Participants were instructed to place the sensors 1–2 m above the floor in a representative area that was away from windows, doorways, the kitchen, and other potential sources of indoor air pollution. Similarly, outdoor sensors were placed 1–2 m above ground level in an area that avoided other pollution sources such as exhaust vents or idling vehicles.

After in-home placement of the sensors, participants turned on their Wi-Fi hotspot to begin real time PurpleAir data transmission to an online database. Data alerts would notify study personnel via email if a sensor went offline, and participants were then contacted to perform basic troubleshoot to get the sensors back online.

Since the optical air pollution sensors in the PurpleAirs typically over-predict PM_2.5_ concentrations, we developed a correction equation using data from 2 PurpleAir sensors that were collocated with a BAM reference monitor in Missoula for the duration of the study. Methods describing the development of the correction equation have been published previously [16].

### 2.3. Survey administration

Study surveys were administered electronically using a platform called Research Electronic Data Capture (REDCap; Vanderbilt University and NIH, USA). We used a private dashboard at the University of Montana to email and track surveys for each participant throughout the study. The surveys and the administration process have been described previously [16].

Following in-home equipment setup, participants completed a baseline survey on demographics and household characteristics. Surveys were modified from those used in previous indoor air quality studies among our research group among similar populations [39–42]. After the baseline survey, participants completed up to 16 weekly surveys that included questions on activity frequency since the previous survey and an in-home BP measurement. Questions for the activity survey were modified from the Yale Physical Activity Survey [43]. A link to complete the weekly survey was emailed to all active participants each Tuesday afternoon. We asked participants to take their BP at a consistent day and time each week (Tuesday evenings at 7pm) and submit SBP and diastolic BP (DBP) results on their weekly electronic survey. Participants were instructed to rest in a seated position for 5 min prior to each measurement. During the measurement, participants were instructed to remain in a seated position with legs uncrossed, feet flat on the floor, and arms relaxed with the BP cuff at approximately the level of their heart.

### 2.4. Wildfire day definition

We used a multi-step process to specify a Wildfire Smoke Day during our study. As we have described previously [44], there is not a standard method for how to define a wildfire smoke event. Various studies have handled this problem differently, largely depending on the type and availability of data within each study. We have based our definition of a Wildfire Smoke Day on previous work from our group [44] and other studies with similar timeframes or data availability [45, 46]. Specifically, this process utilized the study outdoor PurpleAir monitors placed at each participant residence, as well as smoke plume data from the National Oceanic and Atmospheric Administration’s Hazard Mapping System (HMS). HMS data are categorized into daily smoke levels of light, medium, and heavy based on opacity of satellite imagery [47].

For our analysis, we first calculated daily mean outdoor PM_2.5_ concentrations combined across all participants’ outdoor PurpleAir monitors. We then visually inspected HMS maps of the study area for each day with mean PM_2.5_ >10 *µ*g m^−3^ to confirm that the area was impacted by smoke plumes (see supplement for HMS maps) [48]. We used 10 *µ*g m^−3^ as a threshold as this was the approximate PM_2.5_ equivalent for the lower boundary of a medium smoke plume in prior versions of the HMS product [47]. Eighteen study days had mean outdoor PM_2.5_ concentrations of >10 *µ*g m^−3^. Fifteen of these 18 d occurred consecutively during the period from 2–16 September 2022. The other days with mean outdoor PM_2.5_ >10 *µ*g m^−3^ were August 20, October 10, and 20 October 2022. All but three of the days with mean outdoor PM_2.5_ >10 *µ*g m^−3^ had either medium or heavy smoke plumes covering the study area (September 16, October 10, and October 20; see supplement).

After confirming smoke-impacted days according to HMS data, we calculated mean daily PM_2.5_ concentrations for individual participant residences using outdoor PurpleAir data. We then extracted days with household-level PM_2.5_ >21 *µ*g m^−3^, which approximately corresponds to the lower boundary of a heavy smoke plume in prior version of the HMS product [47]. Others, including our group, have used 21 *µ*g m^−3^ daily mean PM_2.5_ to define a wildfire smoke day in previous studies [16, 45, 46]. Specific to HMS data, using heavy smoke days gives further confidence that smoke plumes are impacting ground-level PM_2.5_, whereas lighter smoke plumes detected through satellite imagery can often be less representative of ground-level exposures [46, 47]. Using this 21 *µ*g m^−3^ threshold, we found that 212 of 215 d with PM_2.5_ >21 *µ*g m^−3^ at the household level all occurred between 2–15 September 2022. The other 3 d with PM_2.5_ >21 *µ*g m^−3^ occurred on 7–9 October 2022. According to HMS maps, these dates were impacted by light smoke plumes, and it is possible the elevated PM_2.5_ was from either prescribed burns or other localized sources of air pollution such as wood heating stoves.

Following this analysis, our definition of a Wildfire Smoke Day is days in which the study region is impacted by medium or heavy smoke plumes according to HMS data (2–15 September 2022) and which had household-level daily mean PM_2.5_ concentrations >21 *µ*g m^−3^ according to participant PurpleAir data.

### 2.5. Statistical analysis

All data cleaning and analyses were performed using R software version 4.3.1 [49]. We calculated descriptive statistics for numeric variables (n, mean, standard deviation [sd], minimum [min], 25th percentile [P25], median, 75th percentile [P75], maximum [max]) and categorical variables (n, percent of total [%]). We also summarized SBP and DBP for all study days and separately for wildfire days and non-wildfire days.

Our primary analysis utilized two modeling frameworks to assess associations between air pollution exposures and SBP and DBP. In the first framework, the primary exposure variable was household-level indoor or outdoor PM_2.5_ concentrations (in separate models) from the study PurpleAir sensors. Specifically, the exposure variable was mean PM_2.5_ between weekly survey submissions for each participant. Results from this framework are presented as change in BP per 10 *µ*g m^−3^ increase in mean PM_2.5_. In the second framework, the primary exposure was a categorical variable describing the number of wildfire days in a given survey period for each participant. The wildfire day variable had 3 levels, including 0 (reference level), 1–3, and 4+ wildfire days that occurred during a survey period. The cut points for the wildfire day variable were chosen based on the distribution of the data in order to avoid small numbers in the analysis and have a similar number in each category. Results from this framework are presented as difference in BP compared to the reference level of 0 wildfire days in a survey period.

We used linear mixed models to assess associations between air pollution exposures and BP. Models were adjusted for potential time-variant confounding variables including temperature, relative humidity, and self-reported exercise/activity. Models also included a random-intercept term for participant to account for repeated measures and potential time-invariant confounders. In a secondary model we included additional variables for time of day of BP measurement rounded to the nearest hour, current use of BP medications, and potential time-invariant confounders including participant sex, age, and education. We assessed model assumptions and diagnostic plots for each linear regression model. We have included equations for each modeling framework and variable definitions in the Supplement.

In addition to the primary modeling frameworks, we have conducted secondary analyses to further understand the associations between wildfire exposures and BP. In one analysis, we have combined the two primary modeling frameworks described above. Specifically, the model estimates are the change in BP per 10 *µ*g m^−3^ increase in PM_2.5_ within different levels of the wildfire day variable (0, 1–3, 4+, or any wildfire days). In a further analysis, we have defined a wildfire period as having a wildfire day on the day of the BP measurement, as well as on both days prior to the BP measurement. Model estimates are the difference in BP following a wildfire period compared to a non-wildfire period.

## 3. Results

### 3.1. Participant characteristics

The 20 participants had a mean age of 49 years (sd = 16) and were largely female (*n* = 17, 85%), White (*n* = 20, 100%), and highly educated (*n* = 10 or 50% had a Master’s degree or higher) (table 1). Fifty percent of the participants (*n* = 10) were employed 40 h or more per week and 4 (20%) were retired. A majority of the participants either lived with 1 other person in their household (*n* = 12, 60%) or by themselves (*n* = 2, 10%). Current smoking of any kind was part of the exclusion criteria for the study, and 18 (90%) of the participants reported they had never smoked. Of the 2 participants who had smoked previously, 1 (5%) smoked for less than 1 year and 1 (5%) for 1–5 years. Four participants (20%) reported having a history of high low-density lipoprotein cholesterol, and 3 (15%) reported a history of hypertension and were on BP medications at the time of the study. Fifteen percent of the participants (*n* = 3) also reported having a history of each of the following: heart murmur, low high-density lipoprotein cholesterol, and thyroid disease. One participant (5%) reported history of type-2 diabetes.

**Table 1.** Demographic and health history characteristics among 20 study participants in Western Montana, 2022.

Participant or household characteristic	Summary statistic (*N* = 20)
Age in years, mean (sd), median (min, max)	49 (16), 43 (29, 80)
Unknown, *n* (%)	3 (15)

Sex	
Female, *n* (%)	17 (85)
Male, *n* (%)	3 (15)

Race	
White, *n* (%)	20 (100)
Other, *n* (%)	0 (0)

Ethnicity	
Not Hispanic, *n* (%)	19 (95)
Unknown, *n* (%)	1 (5)

Household income, USD	
<$20 000, *n* (%)	0 (0)
$20 000 to $34 999, *n* (%)	2 (10)
$35 000 to $49 999, *n* (%)	2 (10)
$50 000 to $74 999, *n* (%)	4 (20)
$75 000 to $99 999, *n* (%)	4 (20)
$100 000+, *n* (%)	7 (35)
Unknown, *n* (%)	1 (5)

Education	
High school or less, *n* (%)	0 (0)
Some college, no degree, *n* (%)	1 (5)
Bachelor’s degree, *n* (%)	9 (45)
Master’s degree, *n* (%)	6 (30)
Doctorate or professional degree, *n* (%)	4 (20)

Employment	
Up to 39 h per week, *n* (%)	2 (10)
40 or more hours per week, n (%)	10 (50)
Retired, *n* (%)	4 (20)
Self-employed, *n* (%)	3 (15)
Other, *n* (%)	1 (5)

Total residents living in household	
1, *n* (%)	2 (10)
2, *n* (%)	12 (60)
3, *n* (%)	3 (15)
4, *n* (%)	2 (10)
Unknown, *n* (%)	1 (5)

Smoking history	
Never smoker, *n* (%)	18 (90)
Ever smoker, *n* (%)	2 (10)
Cigarette use less than 1 year, *n* (%)	1 (5)
Cigarette use 1–5 years, *n* (%)	1 (5)

Health history	
Asthma, *n* (%)	2 (10)
Abnormal heart rhythm, *n* (%)	1 (5)
Heart murmur, *n* (%)	3 (15)
Hypertension, *n* (%)	3 (15)
Current blood pressure medications, *n* (%)	3 (15)
High low-density lipoprotein cholesterol, *n* (%)	4 (20)
Low high-density lipoprotein cholesterol, *n* (%)	3 (15)
Circulation problems, *n* (%)	1 (5)
Type 2 diabetes, *n* (%)	1 (5)
Thyroid disease, *n* (%)	3 (15)
Anemia, *n* (%)	1 (5)

sd = standard deviation; USD = United States Dollars; min = minimum; max = maximum.

### 3.2. Participant BP and associations with air pollution

Summary statistics for BP for the entire study and separately for wildfire and non-wildfire periods are presented in table 2. The 20 participants submitted 316 BP measurements over the course of the study, with a mean of 14 BP measurements per participant (range: 9–16). Mean (sd) SBP and DBP over the entire study were 113.5 mmHg (14.5) and 75.4 mmHg (8.8), respectively. Mean SBP was higher during the wildfire period (117.8 mmHg) compared to the non-wildfire period (113.2 mmHg). Mean DBP was also higher during the wildfire period (77.4 mmHg vs 75.2 mmHg).

**Table 2.** Summary statistics for blood pressure among 20 participants in Western Montana, June through October 2022.

Outcome	All study days (n = 2,328)	Wildfire days (*n* = 212)	Non-wildfire days (*n* = 2,116)
	n		
	mean (standard deviation)		
	25th percentile, median, 75th percentile		
Systolic blood pressure, mmHg	316	32	251
	113.5 (14.5)	117.8 (17.0)	113.2 (14.5)
	104, 112, 122	107, 117, 126	103, 111, 122

Diastolic blood pressure, mmHg	316	32	251
	75.4 (8.8)	77.4 (9.5)	75.2 (8.8)
	69, 74, 81	68, 79, 83	69, 74, 80

**Figure 1.**
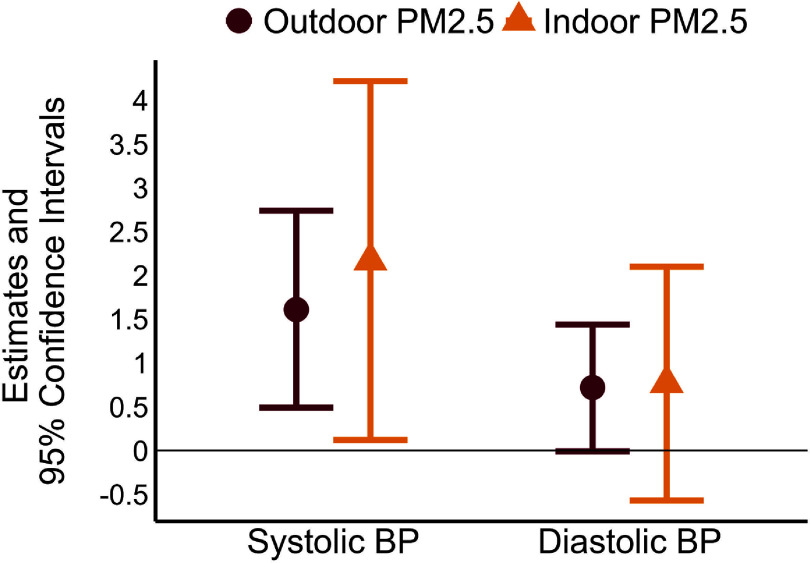
Associations between indoor and outdoor fine particulate matter and in-home blood pressure among 20 participants in Western Montana, June through October 2022. PM_2.5_ = fine particulate matter; BP = blood pressure. Results are from linear mixed models with SBP or DBP as the outcome and PM_2.5_ since the previous health assessment as the exposure. Estimates are expressed as change in blood pressure per 10 *µ*g m^−3^ increase in PM_2.5_. Models include fixed terms for potential time-variant confounders including temperature, humidity, and self-reported indoor and outdoor exercise since the previous health assessment. Models also include a random term for participant to account for repeated measures.

Associations between mean indoor and outdoor PM_2.5_ since the previous health assessment and BP are presented in table 3 and figure 1. Over the entire study, a 10 *µ*g m^−3^ increase in outdoor PM_2.5_ was associated with a 1.34 mmHg increase in SBP (95% Confidence Interval [CI]: 0.39, 2.29) and a 0.71 mmHg increase in DBP (95% CI: 0.07, 1.35). Associations were similar between indoor PM_2.5_ concentrations and BP, although with wider confidence intervals (table 3, figure 1). Results from secondary models were similar to the primary models, with somewhat higher estimates for SBP and similar estimates for DBP (table 3).

**Table 3.** Associations between fine particulate matter and blood pressure among 20 participants in Western Montana, June through October 2022.

Outcome	Outdoor PM_2.5_ (*µ*g m^−3^)		Indoor PM_2.5_ (*µ*g m^−3^)	
	n	Estimate (95% CI)	n	Estimate (95% CI)
Systolic blood pressure, mmHg (primary model)	272	1.34	266	1.48
		(0.39, 2.29)		(−0.28, 3.24)

Systolic blood pressure, mmHg (secondary model)	231	1.61	225	2.17
		(0.49, 2.74)		(0.12, 4.22)

Diastolic blood pressure, mmHg (primary model)	271	0.71	265	0.77
		(0.07, 1.35)		(−0.44, 1.97)

Diastolic blood pressure, mmHg (secondary model)	230	0.72	224	0.77
		(−0.01, 1.44)		(−0.57, 2.10)

PM_2.5_ = fine particulate matter; CI = confidence interval; *n* = number of observations in the model.

Results are from linear mixed models with SBP or DBP as the outcome and PM_2.5_ since the previous health assessment as the exposure. Estimates are expressed as change in BP per 10 *µ*g m^−3^ increase in PM_2.5_. Models include fixed terms for potential time-variant confounders including temperature, humidity, and self-reported indoor and outdoor exercise since the previous health assessment. Models also include a random term for participant to account for repeated measures. Secondary models also include fixed terms for time of day of BP measurement rounded to the nearest hour, current use of BP medications, and potential time-invariant confounders including participant sex, age, and education.

**Figure 2.**
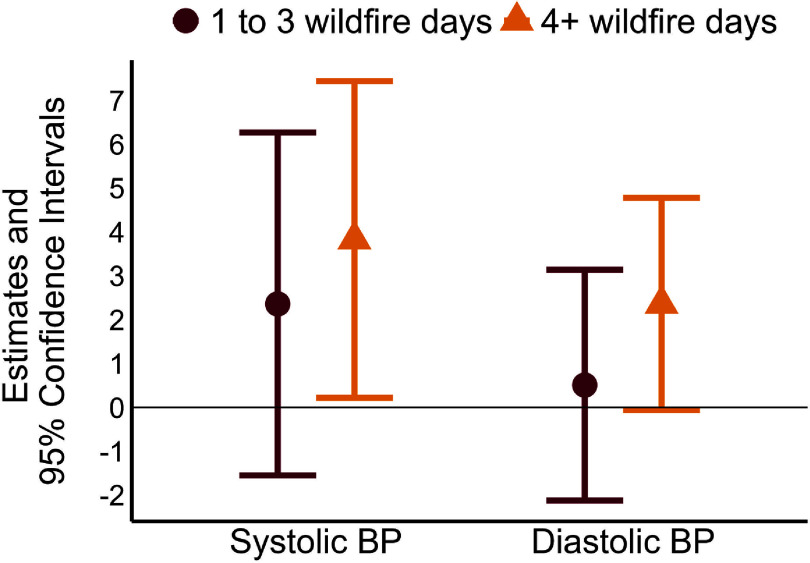
Associations between wildfire vs non-wildfire impacted periods and in-home blood pressure among 20 participants in Western Montana, June through October 2022. BP = blood pressure. Results are from linear mixed models with SBP or DBP as the outcome and wildfire day classification as the exposure. Exposure levels indicate the number of wildfire days since the previous BP measurement. Estimates are expressed as difference in BP compared to the reference level of zero wildfire days. Models include fixed terms for potential time-variant confounders including temperature, humidity, and self-reported indoor and outdoor exercise since the previous health assessment. Models also include a random term for participant to account for repeated measures.

Associations between wildfire vs non-wildfire impacted periods and BP are presented in table 4 and figure 2. SBP and DBP were higher during periods with 1–3 wildfire days compared to non-wildfire periods, although confidence intervals were wide and crossed 0 (SBP estimate: 2.36 mmHg; 95% CI: −1.55, 6.27; DBP estimate: 0.51 mmHg; 95% CI: −2.12, 3.14). Associations were stronger during periods with 4+ wildfire days compared to non-wildfire periods. For example, SBP was 3.83 mmHg higher (95% CI: 0.22, 7.44) and DBP was 2.36 mmHg higher (95% CI: −0.06, 4.78) during periods with 4+ wildfire days compared to non-wildfire periods. Results from secondary models were similar to the primary models, although with somewhat higher estimates for periods with 4+ wildfire days and lower estimates for periods with 1–3 wildfire days (table 4).

**Table 4.** Blood pressure during wildfire vs non-wildfire periods among 20 participants in Western Montana, June through October 2022.

Outcome	Exposure	n	Estimate (95% CI)
Systolic blood pressure, mmHg (primary model)	Non-wildfire period	222	Reference
	1–3 wildfire days	21	2.36 (−1.55, 6.27)
	4+ wildfire days	29	3.83 (0.22, 7.44)

Systolic blood pressure, mmHg (secondary model)	Non-wildfire period	189	Reference
	1–3 wildfire days	16	0.33 (−4.53, 5.19)
	4+ wildfire days	26	4.95 (0.75, 9.15)

Diastolic blood pressure, mmHg (primary model)	Non-wildfire period	221	Reference
	1–3 wildfire days	21	0.51 (−2.12, 3.14)
	4+ wildfire days	29	2.36 (−0.06, 4.78)

Diastolic blood pressure, mmHg (secondary model)	Non-wildfire period	188	Reference
	1–3 wildfire days	16	0.15 (−2.95, 3.26)
	4+ wildfire days	26	2.80 (0.12, 5.48)

CI = confidence interval; *n* = number of observations in the model.

Results are from linear mixed models with SBP or DBP as the outcome and wildfire day classification as the exposure. Estimates are expressed as difference in BP compared to reference. Models include fixed terms for potential time-variant confounders including temperature, humidity, and self-reported indoor and outdoor exercise since the previous health assessment. Models also include a random term for participant to account for repeated measures. Secondary models also include fixed terms for time of day of BP measurement rounded to the nearest hour, current use of BP medications, and potential time-invariant confounders including participant sex, age, and education.

Results in table S1 indicate that the associations between PM_2.5_ and BP from table 3 are largely driven by PM_2.5_ exposures during wildfire periods. For example, a 10 *µ*g m^−3^ increase in PM_2.5_ during a period with any wildfire days was associated with a 1.85 mmHg increase in SBP (95% CI: −0.09, 3.79), whereas a 10 *µ*g m^−3^ increase in PM_2.5_ during a period with no wildfire days was not associated with a meaningful change in SBP (estimate = − 1.97; 95% CI: −7.92, 3.99). From table S2, results with an alternate wildfire period definition (wildfire day on the day of the BP measurement and both days prior) had very similar results to periods with 4+ wildfire days (table 4).

## 4. Discussion

In this study of adults in Western Montana during the summer of 2022, we found associations between air pollution exposures and BP during the study using 2 analytical frameworks. A 10 *µ*g m^−3^ increase in outdoor PM_2.5_ was associated with a 1.34 mmHg increase in SBP and a 0.71 mmHg increase in DBP. We also found that SBP was 3.83 mmHg higher and DBP was 2.36 mmHg higher during survey periods with 4+ wildfire days compared to non-wildfire periods. As wildfires continue to increase under climate change, these results are important contributions to the literature on the cardiovascular health impacts of wildfire air pollution.

Although there is extensive literature on the cardiovascular health effects of ambient air pollution exposures [11–13], including impacts on BP specifically [10], much of the existing research has been related to ambient air pollution exposures in urban environments that are largely from traffic and industrial sources. In a meta-analysis of associations between ambient air pollution and BP by Yang *et al* (2018), results from 30 studies on short-term effects of PM_2.5_ exposure showed a 0.53 mmHg increase in SBP (95% CI: 0.26, 0.80) and a 0.20 mmHg increase in DBP (95% CI: 0.02, 0.38) per 10 *µ*g m^−3^ increase in PM_2.5_ [10]. Effects from 36 studies on long-term PM_2.5_ exposure were less consistent, with a non-significant 0.37 mmHg increase in SBP (95% CI: −0.65, 1.39) and a 0.47 mmHg increase in DBP (95% CI: 0.12, 0.82) per 10 *µ*g m^−3^ increase in PM_2.5_ [10]. By comparison, the associations we observed between short-term PM_2.5_ and BP in a rural, mountainous region during wildfire season were 2–3 times larger than the overall association between short-term PM_2.5_ and BP in the meta-analysis. However, it should also be noted that there was high heterogeneity in the study designs used in the meta-analysis, and the associations we have reported in tables 3 and S1 fall within a similar range of many of the individual studies in the meta-analysis. There are also associations between biomass air pollution exposures and BP from controlled exposure studies with both human and animal models [50–52], as well as studies on the use of wood fuels for cooking and heating [53–56].

In contrast, few studies have assessed the impact of wildfire-emitted air pollution exposures on BP due to the numerous logistical constraints of conducting field studies during wildfire events. Wildfires are difficult to predict, and field studies are logistically burdensome to conduct. As such, most of the previous studies on wildfire health impacts have been epidemiologic in nature, utilizing exposures from air pollution models or stationary monitors and clinical outcomes from electronic health records [29]. A study during the Athens, Greece wildfires of 2021 assessed in-home BP and PM_2.5_ among 20 adults under treatment for hypertension [57]. The authors reported a 1.3 mmHg increase in SBP per 1 *µ*g m^−3^ increase in ambient PM_2.5_ from the nearest stationary monitor after adjusting for smoking status [57]. To our knowledge, ours is the only other study to date to assess wildfire air pollution and in-home BP. While our results are smaller in magnitude (our associations are per 10 *µ*g m^−3^ increase in PM_2.5_, consistent with the Yang *et al* meta-analysis), our study also implemented more robust, household-level exposure assessment with longer follow-up [16]. Our findings from table S1 also suggest stronger associations between PM_2.5_ and BP during periods impacted by wildfire compared to associations throughout the entire study. Other differences in health assessments, study population, composition of the air pollution during the studies, and study design all could have led to the different magnitude of the results of these studies. Overall, however, both studies indicate an adverse association between wildfire air pollution exposures and SBP.

Wildfires are increasing globally and will continue to worsen under climate change [20, 22, 23]. Air pollution from biomass sources such as wildfires, as opposed to ambient air pollution from other sources, has a different chemical composition of pollutants and may have differential impacts on health [29, 34]. There is also evidence that local wildfire smoke and long-range transport smoke from distant fires may have differential impacts on health [26]. As wildfires increase and make up a larger proportion of ambient air pollution [22, 23], it is crucial that we understand the impact on public health. BP as an outcome is particularly important due to the global burden of hypertension and the impact on cardiovascular disease progression [5]. While acute clinical outcomes such as myocardial infarction are important, BP is a representation of underlying, chronic cardiovascular disease. By understanding the impact of wildfires on BP, we can emphasize the importance of public health interventions to more consistently reduce the adverse health impacts of wildfire air pollution.

Another strength of our study that is crucial to understanding the impact of wildfire smoke and intervention strategies is that we measured both indoor and outdoor PM_2.5_ at each participant’s household. Associations between BP and indoor and outdoor PM_2.5_ were similar in this study (table 3), further demonstrating a need for intervention strategies that can improve indoor air quality and subsequent health outcomes. In a systematic review and meta-analysis of 10 trials assessing the impact of indoor air filtration on BP, Walzer *et al* reported that use of portable air cleaners (PACs) to filter indoor air led to 3.94 mmHg lower SBP and 0.95 mmHg lower DBP among over 600 participants with a median follow-up of 13.5 d [58]. A more recent study in New York City assessed the impacts of reduction in PM_2.5_ through air filtration on in-home BP. Authors reported that air filtration had a non-significant reduction in SBP (129.6 mmHg for filtration arm vs 134.6 mmHg for sham arm) [59]. Given the impact that air filters and other behaviors can have on reducing exposure to PM_2.5_ and lowering BP, clinicians can play an important role by educating patients with existing cardiovascular disease on the importance of air pollution exposures and advise on behaviors and strategies for reducing exposures [31, 60]. Clinical researchers have provided a potential framework through screening for air pollution exposures and outlining targeted intervention strategies that can be evaluated in future studies to reduce exposures to air pollution and subsequently improve health [31, 60].

Although our sample size of 20 participants is small, a strength of our study was the repeated measures of up to 16 in-home BP measurements per participant over the course of an entire wildfire season. The generalizability of our results may also be limited, as our participants were recruited from a local climate advocacy organization and mostly identified as white and female. However, our findings in this pilot study highlight the feasibility of a distance-based field study with household-level PM_2.5_ measurements and subclinical health outcomes. It is also important to emphasize that our study population was made up of generally healthy adults with few comorbidities and only 3 participants with diagnosed hypertension (table 1). It is possible that associations between wildfire air pollution and BP could be different among a more clinically susceptible population, and our study framework will be applied to more representative and vulnerable populations in future research. Finally, the 2022 wildfire season in Montana was relatively mild compared to other recent years, and only around 2 weeks of the 4 month study were impacted by wildfire smoke. Our previously published work from this study highlights the contrast in PM_2.5_ during wildfire versus non-wildfire periods [16]. During non-wildfire periods, mean 24 h outdoor PM_2.5_ across all study households was 3.6 *µ*g m^−3^ (sd = 5.2, median = 2.1). In contrast, mean 24 h outdoor PM_2.5_ across all study households during the wildfire period was 15.9 *µ*g m^−3^ (sd = 14.7, median = 10.4). Using these data, we were also able to assess associations with BP during the distinct wildfire and non-wildfire periods (table S1). This analysis and the results from table S1 show that the associations between PM_2.5_ and BP from table 3 are largely driven by PM_2.5_ from the wildfire periods and that there was no meaningful association between PM_2.5_ and BP during the non-wildfire periods. Although it is possible associations would be different in a study with higher wildfire smoke exposures, our results also highlight the importance of our distance-based methods. Even in a mild wildfire year, our novel methods allowed us to conduct this research in a field setting to better understand the subclinical health impacts of wildfires.

## 5. Conclusion

Our results add to the growing body of evidence that wildfire air pollution exposures adversely impact health outcomes including BP. Given the increasing threat of wildfires under climate change, and the global burden of BP and hypertensive disorders on cardiovascular disease and mortality, our findings highlight the importance of improved messaging on the health impacts of wildfires and effective strategies to reduce exposures to wildfire smoke. Such strategies are particularly important for clinically susceptible populations, such as those with existing cardiovascular disease. Clinicians and public health professionals alike can play an important role in delivering education and practical recommendations such as utilizing air filters to improve indoor air quality.

## Data Availability

The data cannot be made publicly available upon publication because they contain sensitive personal information. The data that support the findings of this study are available upon reasonable request from the authors.
